# The comparative evidence for urban species sorting by anthropogenic noise

**DOI:** 10.1098/rsos.172059

**Published:** 2018-02-21

**Authors:** Gonçalo C. Cardoso, Yang Hu, Clinton D. Francis

**Affiliations:** 1 CIBIO—Centro de Investigação em Biodiversidade e Recursos Genéticos, Universidade do Porto, Campus Agrário de Vairão, 4485-661 Vairão, Portugal; 2 Behavioural Ecology Group, Department of Biology, University of Copenhagen, 2100 Copenhagen Ø, Denmark; 3 School of BioSciences, University of Melbourne, Melbourne, Victoria 3010, Australia; 4 Department of Biological Sciences, California Polytechnic State University, San Luis Obispo, CA, USA

**Keywords:** anthropogenic noise, bird song, sound frequency, urban ecology, noise-filter hypothesis

## Abstract

Anthropogenic noise is more intense at lower sound frequencies, which could decrease urban tolerance of animals with low-frequency vocalizations. Four large comparative studies tested whether anthropogenic noise filters bird species according to the sound frequencies they use and produced discrepant results. We reanalysed data from these studies to explain their different results. Urban tolerance of bird species (defined here as often occurring and breeding in cities) is very weakly related to urban preference or relative abundance (defined based on changes in population density from urban to nearby rural environments). Data on urban preference/abundance are potentially accurate for individual cities but differ among cities for the same species, whereas existing data on urban tolerance are coarser but provide a more global synthesis. Cross-species comparisons find a positive association between the sound frequency of song and urban tolerance, but not urban preference/abundance. We found that showing an association between song frequency and urban tolerance requires controlling for additional species traits that influence urban living. On the contrary, controlling for other species traits is not required to show a positive association between song frequency and use of noisy relative to quiet areas within the same type of environment. Together, comparative evidence indicates that masking by urban noise is part of a larger set of factors influencing urban living: all else being equal, species with high-frequency sounds are more likely to tolerate cities than species with low-frequency sounds, but they are not more likely to prefer, or to be more abundant in, urban than non-urban habitats.

## 1. Background

Anthropogenic noise, mostly due to motorised traffic, is pervasive in cities and near main roads. It can affect communication by masking animal acoustic signals [1], and negatively impact the abundance (e.g. [2–6]) and fitness [7–9] of some animals in noisy environments. Because urban noise is most intense at low sound frequencies, it has been suggested that animals using acoustic signals with higher sound frequencies would be less affected by noise and, thus, more tolerant of urban environments. This idea was coined as the urban noise-filter hypothesis [10].

Four large-scale comparative studies tested the urban noise-filter hypothesis, all using birds, but with discrepant results. Two of these studies [11,12] assessed urban tolerance based on whether species are described as commonly occurring in urban areas, and the other two [10,13] used comparisons of population densities across habitats. These approaches may have different biological meanings: the presence/absence approach in the former studies assessing the ability to occur and breed in cities, and the approach based on comparing population densities (in urban or noisy habitats versus non-urban or non-noisy habitats) assessing instead habitat preferences or the abundance in urban or noisy habitats versus others.

The first of these studies, Hu & Cardoso [11], noted which bird species are described in the literature as commonly occurring in cities, and compared their average sound frequency with that of congeneric species not described as inhabiting urban habitats (529 species in 103 genera). On average, species occurring in cities sing (passerines) or vocalize (non-passerines) with higher dominant sound frequency than their non-urban congeners. Using the same classification of occurrence in cities, Cardoso [12] tested for a relation with the dominant sound frequency of songs, song loudness and several ecological traits across 140 passerine species. Higher sound frequency was significantly related to occurrence in cities, with an effect size (*β*_st_ = 0.21, *r*^2^ = 0.03) of about half that for the ecological trait most closely related to urban tolerance (off-ground nesting, *β*_st_ = 0.30, *r*^2^ = 0.06 [12]). Francis [13] reviewed the relative abundance (e.g. occupancy, density, breeding density) for 308 populations of 183 bird species along different gradients of anthropogenic noise, and tested which species traits predict differences in abundance along those noise gradients. Together with other traits (ground foraging and a plant-based diet), high sound frequency was one of the main species traits associated with living in noisy relative to quiet areas (*β* = 0.138 ± 0.03 s.e. [13]). These three studies concur in supporting the urban noise-filter hypothesis. The fourth study, Moiron *et al*. [10], tested for associations between the sound frequency of song and metrics of urban tolerance based on differences in population density within and outside urbanized areas for 384 passerine species. Contrary to the previous comparative studies, and despite the accurate metrics and very good statistical power of this analysis, there were no detectable relations between the sound frequency that the different species use and metrics of urban tolerance.

Moiron *et al*. ([10]; also [14]) suggest that comparing urban with nearby non-urban densities is preferable to using presence/absence data because the absence from some cities can simply be due to a species not being present in that region and, therefore, not able to disperse to or establish in those cities. This can be important, especially when distance to urban areas limits dispersal to and production of emigrants that could establish in cities. But urban bird species tend to be good dispersers [15], suggesting that, for urban-tolerant species, urban establishment may occur even if source populations are not nearby. When dispersal is not limiting, a species may achieve similar urban densities irrespective of being common or rare in nearby non-urban populations, and accounting for nearby non-urban densities may even be detrimental in some cases. Additionally, if urban tolerance varies within a species (e.g. it is urban tolerant only in some geographical regions), accurately assessing response to urbanization in a given region may fail to capture how urban tolerant that species is globally, which is perhaps better assessed by coarser but more global classifications based on habitat descriptions in the literature.

Both Francis [13] and Moiron *et al*. [10] used abundance data to assess population responses to anthropogenic noise or urbanization, respectively. The different results in these two studies may be due to Moiron *et al*. [10] studying gradients of urbanization, while Francis [13] studied gradients of anthropogenic noise that are not necessary in urban areas (e.g. proximity of roads or industry). Another difference between studies is that Moiron *et al*. [10] controlled for two species traits most likely to be associated with sound frequency (body size and type of habitat), while Francis [13] and Cardoso [12] controlled for various additional species traits (e.g. nest type, diet, ground dwelling or foraging). Also, Hu & Cardoso [11] restricted their pair-wise comparisons to species within the same genera, which very efficiently minimizes variation in confounding traits because congeneric species usually have similar ecology and morphology. Accounting for confounding factors might also help explain the different results of these studies.

Here we reanalyse data from these comparative studies to evaluate the standing of the comparative evidence for the urban noise-filter hypothesis. For clarity, hereafter we use the term urban tolerance to refer to metrics based on the presence/absence data, and the terms urban preference or abundance for metrics that compare population densities in urban versus non-urban environments. We ask whether urban tolerance (inferred from habitat descriptions, as generally occurring or not occurring in urban areas) and urban preferences (inferred from comparing density data in urban and non-urban areas) are similar or different, and we assess how much of the difference between the two approaches is due to geographical variation in urban tolerance. We also reanalyse the data of Cardoso [12] and Francis [13] to determine whether the reported associations between sound frequency and living in urban or noisy areas require controlling for confounding traits.

## 2. Assessments of urban tolerance and urban preference

There are 365 species common to the dataset of Hu & Cardoso [11] (including species not included in paired comparisons because of lack of within-genera differences in using urban environments) and the dataset of Sol *et al*. [16] for *tolerance class*, which is the main metric used by Moiron *et al*. [10]. The metric *tolerance class* is based on simulation tests of whether the urban versus non-urban densities differ from a random dispersal model [10,14,16] and, for that reason, it is more robust to inaccuracies of insufficient censusing than simpler descriptive metrics (D Sol 2015, personal communication). For the 365 species common to these two datasets, we correlated the habitat classifications of Hu & Cardoso [11] (1, not occurring, 1.5, occurring infrequently or 2, occurring in urban environments) with *tolerance class* (1, avoider, 1.5, neutral or 2, exploiter of urban environments, averaged across multiple cities when a species was studied in more than one city; data from table S7 of [16]). The correlation between the two was significant but very low (*r* = 0.12, *p* = 0.02; dataset in electronic supplementary material, table S1). Therefore, one or both of these metrics provide a poor indication of species-typical urban tolerance, and/or they assess distinct aspects of how birds react to urbanization.

To understand the low correlation between the two metrics, we first assessed the repeatability (as in [17]) of *tolerance class* for the 215 species in the dataset of Sol *et al*. [16] that were studied in more than one city (average 3.14 cities, range from 2 to 9). The repeatability of *tolerance class* within species across cities was significant but low (0.29, *p* < 0.001). When calculated with the data from all the 365 species common to the two datasets above (average 2.08 cities per species, range from 1 to 9), repeatability of *tolerance class* is similarly low (0.26, *p* < 0.001). There is, therefore, substantial variation among cities in how population densities of the same bird species change with urbanization. These differences in the estimates of *tolerance class* for the same species in different cities may be in part methodological (e.g. differences among cities in sample sizes and statistical power, or in the suitability of surrounding habitats), but nonetheless suggest that some bird species may differ geographically in urban tolerance. For example, there appears to be a higher proportion of urban-tolerant passerine species in Europe than North America [12], perhaps because denser and older urbanization in Europe enhanced the opportunity for urban adaptation. The large within-species variation in *tolerance class* places an upper ceiling on its correlation with more global metrics of how species react to urbanization because, for example, the assessment of *tolerance class* for a given species may depend on the actual cities included in the study.

We calculated the effect that the within-species variation in *tolerance class* has in setting an upper ceiling on its correlation with other metrics, using data from the 365 species common to the datasets of Hu & Cardoso [11] and Sol *et al*. [16], and the simulations described in appendix A. Simulations showed a value of 0.57 (95% CI: 0.50 to 0.64) as the maximum correlation coefficient possible between local estimates of *tolerance class* and their underlying, species-typical values. Therefore, within-species variation in *tolerance class* prevents high correlations with global metrics of urban tolerance. This should contribute to the low correlation between *tolerance class* and the habitat classifications that Hu & Cardoso [11] used to assess urban tolerance. But the correlation between these two metrics (*r* = 0.12, see above) is still very low compared to the upper ceiling calculated here (about one-fifth of the ceiling), meaning that (1) habitat classifications in Hu & Cardoso [11] also have substantial inaccuracy and/or (2) the two metrics assess distinct aspects of the response to urbanization. It is useful to consider how each of these two scenarios can affect testing the association between sound frequency and urban tolerance.

(1) Hu & Cardoso ([11]; also [12]) classified habitat descriptions into a three-level scale of urban occurrence (not occurring, occurring infrequently or occurring). This is a coarse and inaccurate scale that misses much variation in how different species cope with urbanization and, thus, it must have contributed to the low correlation coefficient between the two metrics above. This coarse but global approach is justified by the compromise we showed here between local accuracy and geographical representativeness. A coarse scale can cause more conservative statistical testing, because fine-scale associations between sound frequency and urban tolerance are missed. But, apart from this conservativeness, coarse scales do not bias results in a particular direction. Therefore, scaling issues do not explain why these studies [11,12] found higher sound frequency in urban-tolerant species.

(2) *Tolerance class* and other metrics in Moiron *et al*. [10] are based on population densities in urban and nearby non-urban areas, and thus quantify urban preference versus avoidance (or the overall abundance in cities versus non-urban areas). Instead, approaches based on occurrence versus non-occurrence in urban areas, such as in Hu & Cardoso ([11]; also [12]), assess the ability to inhabit and breed in urban areas, irrespective of whether urban or non-urban areas are preferred. The difference in the two approaches is apparent comparing scorings on the same species (electronic supplementary material, table S1). For example, among species observed by Sol *et al*. [16] in more than five cities (first 23 species in electronic supplementary material, table S1), most species (19 of 23) are classified by Hu & Cardoso [11] as occurring in urban areas, but most of those species (17 of 23) are scored by Sol *et al*. [16] below the median for *tolerance class*, including several iconic urban dwellers (e.g. European blackbird, blue and great tits). These discrepancies are excessive to attribute only to lack of accuracy in either classification, and instead suggest that the two approaches target distinct aspects of how species react to urbanization.

Thus, while *tolerance class* and other metrics based on population densities assess the preference for, or abundance in, cities, approaches based on presence or absence assess the ability or inability to cope with urban environments. The latter is a coarser approach, but is closer to the meaning of tolerance as opposed to preference. Using *tolerance class*, Sol *et al*. [16] found that some species traits, related to life history and resource use, predict the abundance of bird species in cities. This is sensible because species that best use ecological opportunities offered by cities (e.g. anthropogenic food resources, or protection from certain predators [18,19]) should attain higher urban densities. On the contrary, we do not predict that species using high sound frequencies will achieve higher densities in cities than elsewhere, because they are not using a resource that is enhanced in cities (most non-urban environments also have weak noise in the high frequencies). These species are only predicted not to be excluded from urban areas due to noise, a prediction well captured by the coining of this hypothesis by Moiron *et al*. [10] as the urban noise filter. It is, therefore, sensible that the urban noise-filter hypothesis is supported by comparative studies focusing on the ability to cope with urban environments, rather than on the preference or abundance in those environments.

## 3. Accounting for covariates

The multivariate analysis in Cardoso [12], relating occurrence in urban habitats to various song, morphological and ecological traits across 140 passerine species, found a significant positive association with the dominant sound frequency of songs (hereafter, song frequency; *β*_st_ = 0.21, *F*_130,1_ = 4.85, *p* = 0.03, first line of table 1). To assess if detecting this association requires controlling for confounding factors, here we repeat this analysis each time removing a different song, morphological or ecological covariate (methods in appendix B). Table 1 shows the estimated effect of song frequency on urban occurrence when removing each of these covariates.

**Table 1.** Association of the sound frequency of song and urban occurrence across 140 passerine species using the phylogenetic multiple regression model in Cardoso [12], and identical models lacking one covariate each. *β*_st_, standardized partial regression coefficient. Degrees of freedom of *F* are 130,1 for the original model and 131,1 for the remaining. Estimated *λ* for all models was zero (methods in [12]).

	association of song frequency and urban occurrence		
covariate absent from model	*β*_st_	*F*	*p*-value
none (original model in Cardoso [12])	0.21	4.85	0.03
omnivory	0.22	5.30	0.02
cavity or rock nesting	0.21	4.85	0.03
preferred vegetation density	0.21	4.74	0.03
loudness index	0.20	4.31	0.04
ground nesting	0.18	3.34	0.07
foraging on ground	0.17	3.36	0.07
body mass	0.13	2.26	0.13

Removing species traits related to the type of food, cavity or rock nesting, vegetation density and song loudness, each had small effects on the association between song frequency and urban occurrence. The association may weaken slightly, but always remained significant (second to fifth lines in table 1); removing more than one covariate at a time would cause larger changes. Removal of ground nesting, ground foraging or body mass had larger effects, each enough to cause the relation between song frequency and urban occurrence to no longer be statistically significant (three last lines in table 1). Thus, variation in traits such as body size and the use of ground versus higher strata make it difficult to show the association between song frequency and urban tolerance, and such traits need to be controlled for given their own influence on urban tolerance independently of acoustics.

The multivariate analysis in Francis [13] similarly found that, across species, higher song frequency was associated with resilience to decreases in population density along noise gradients in otherwise similar habitat (*β*_st_ = 0.13; ± 0.03 s.e.). Again, to assess if this result hinges on controlling for covariates, we repeated similar analyses removing a different covariate each time (methods in appendix B). The result was remarkably robust to removing individual covariates. Removing variables related to either song, morphology or ecology, all caused practically no change in the strength of the relationship between song frequency and tolerance to noise (table 2). Together, these reanalyses suggest that associations between song frequency and living in noisy relative to quiet areas (within a similar environment) are less confounded by other species traits than associations with urban tolerance. It is sensible that detecting associations with urban tolerance require controlling for confounding factors, because living in urban environments is influenced by many species traits (e.g. [16]) in addition to those related to noise tolerance.

**Table 2.** Influence of song frequency on responses to noise across 308 populations of 183 bird species, using the model averaging approach of Francis [13] and identical approaches lacking one covariate each. *β*, model-averaged partial regression coefficient (±s.e.).

covariate absent from model	*β ± *s.e.	*z* (*p*-value)	no. averaged models
none (included all covariates with VIS > 0.5)	0.14 ± 0.03	5.01 (<0.001)	33
song interval	0.14 ± 0.03	5.34 (<0.001)	27
song length	0.14 ± 0.03	5.2 (<0.001)	15
body mass	0.14 ± 0.02	5.93 (<0.001)	21
foraging location	0.14 ± 0.02	5.41 (<0.001)	19
nest type	0.14 ± 0.03	5.04 (<0.001)	22
diet	0.14 ± 0.03	4.88 (<0.001)	17
response type	0.14 ± 0.03	5.04 (<0.001)	20

In conclusion, comparative support for the urban noise-filter hypothesis comes either from work looking at noise gradients rather than urbanization gradients [13], or work on urban tolerance (assessed by urban presence or absence) accounting for confounding effects of other species traits [11,12]. Urban tolerance (here defined as the ability to inhabit urban areas, and assessed by the presence or absence there) is very weakly related to urban preference or abundance (assessed by comparing urban and non-urban densities), which is sensible because anthropogenic noise is not an ecological resource that should make noise-tolerant species prefer urban over non-urban environments. Accordingly, species with high-frequency songs do not prefer nor are more abundant in urban versus non-urban areas when compared to species with low-frequency songs [10]. The available comparative data supports the urban noise-filter hypothesis in that, all else being equal, low-frequency species are less likely to inhabit noisy or urban habitats, but also indicates that this filtering is not responsible for differences in urban abundance among the different species that do inhabit cities.

## Supplementary Material

Supplementary Table S1

## Data accessibility

The dataset supporting this article was uploaded as the electronic supplementary material.

## Appendix A

We assessed the maximum possible correlation coefficient between the metric *tolerance class* and more global metrics, based on the within-species variation in *tolerance class* for the 365 species common to the datasets of Hu & Cardoso [11] and Sol *et al*. [16]. For this, we used a two-step simulation procedure. Step 1 simulates global values (i.e. species-typical values) and local estimates (i.e. values in different cities) of *tolerance class* using within-species geographical variation similar to that in the real data. Step 2 then estimates the correlation between the local estimates of *tolerance class* in different cities and the global, species-typical value of *tolerance class* of each species.

Step 1: For 365 virtual species we simulated global, species-typical values of urban tolerance by bootstrapping (i.e. sampling randomly with replacement) from the *tolerance class* scores of Sol *et al*. [16] for the real 365 species (i.e. from the 365 weighted species means across cities in electronic supplementary material, table S1). For each virtual species, we created local estimates of urban tolerance by adding variation as a random number from a normal distribution with standard deviation *x*. The number of local estimates per species varied (from 1 to 9 cities; electronic supplementary material, table S1) to match the number of cities from which the real 365 species were studied in the dataset of Sol *et al*. [16]. We then calculated the within-species repeatability of these local estimates (as in [17]). We repeated this (bootstrapping global values, and creating local estimates) 1000 times, each time computing repeatability. Finally, we computed the mean repeatability across the 1000 repeats. The entire step 1 was repeated iteratively for different values of *x*, until finding the value *x′* that gives a mean repeatability identical to that of *tolerance class* for the real 365 species to the third digit (0.255; see main text). The purpose of these iterations was to calibrate the magnitude of the simulated among-city variation to match that in the real data.

Step 2: We simulated global values and local estimates of urban tolerance as in step 1, only this time using *x′* as the standard deviation of the among-city variation, and then calculated the correlation coefficient between the global value and the average of its local estimates for each of the 365 virtual species. Step 2 was repeated 1000 times to obtain the expected correlation between the local estimates of *tolerance class* and their global, species-typical value (median correlation coefficient across the 1000 repeats) and its 95% CI (2.5th to 97.5th percentile of the distribution of correlation coefficients).

## Appendix B

We repeated the phylogenetic generalized least-squares (PGLS) multiple regression model of Cardoso [12], using the same phylogeny and data as in the original article, but each time removing one of the covariates related to the song, morphology or ecology of the bird species. Only one of the covariates is missing each time; removals are not cumulative. Here we report the effect that each removal has on the estimated relationship between song frequency and urban occurrence (lines 2–8 in table 1), and compare it to the original estimated relationship in the full model (first line in table 1).

Francis [13] reported both PGLS and linear mixed effects models. Here we only use the latter because phylogenetic signal was near zero for these data and accounting for phylogeny is, therefore, superfluous. As in the original paper, we performed model averaging across all well-supported models (models with ΔAIC_c_ ≤ 4 from the best). The first line of table 2 reports the effect of song frequency on responses to noise when analysing all covariates that received variable importance scores greater than 0.5 in at least one of the original analyses (PGLS or mixed effects models; fig. 3 in [13]). The remaining lines in table 2 report the same effect when removing one covariate (again, only one, non-cumulatively) prior to model selection and averaging. We report model-averaged parameter estimates for the influence of song frequency on response to noise (±s.e.), the *z*-test statistic for song frequency on response to noise (*Z* and *P*), and the number of averaged models (i.e. models with ΔAIC_c_ ≤ 4).
